# New Appliances and Things Medical

**Published:** 1897-06-05

**Authors:** 


### NEW APPLIANCES AND THINGS MEDICAL.

[We shall be glad to receive, at our Office, 28 & 29, Southampton Street, Strand, London, W.C., from the manufacturers, specimens of all new preparations and appliances which may be brought out from time to time.]

### HARTMANN'S PATENT WOOD WOOL PREPARATIONS.

To meet the great demand that has arisen for an antiseptic towelette, the Sanitary Wood Wool Company (Limited), 26, Thavie's Inn, Holborn Circus, have recently brought out a most comfortable and healthful article, at sixpence a dozen. They are soft and antiseptic; all risk of infection is avoided; they are invaluable when travelling, as they can easily be burnt; and the initial cost is less than the washing of ordinary towels. We have, therefore, much pleasure in recommending them on all these grounds to the favourable consideration of our readers, and feel sure that they will be found at once a comfort and a boon.

### NEW DIGESTIVE PREPARATIONS.

(Giles, Schacht, and Co., Clifton, Bristol.)

We have had three samples of the above preparations submitted to us, namely, (1) the bi-digestive, (2) bi-digestive tonic, (3) coca bi-digestive. The main point achieved by this new preparation is that diastatic and proteolytic ferments have been combined in one and the same preparation, each able to exercise its own particular function in the presence of the other. The Schacht bi-digestive is a mixture of an agreeable flavour, containing a diastase and a proteolytic ferment. The bi-digestive tonic contains in addition iron, lime, cinchona, and nux-vomica; and the coca bi-digestive contains the active principles of coca, combined with those of pepsine and diastatic malt. In no preparations claiming to operate as adjuvants to digestion that we have hitherto examined have we obtained such satisfactory results, and, as far as our experience goes, the three digestives perform all that is claimed for them by the manufacturers.

### DE KUYPER'S GENEVA HOLLANDS.

(Rotterdam.)

This excellent spirit has been submitted by us to careful clinical tests, and, combined with milk for invalids who require alcoholic stimulant, we find it an exceedingly palatable and acceptable substitute for other forms of spirit. Many invalids who require alcohol in some form object to the distinctive flavour of whisky or brandy, and in such cases we have frequently found that De Kuyper's Hollands can be combined with milk and taken by the patient without any complaint or objection. This spirit is undoubtedly of the greatest purity, of fine quality, and particularly dry, and there are certainly many gouty, rheumatic, and plethoric patients who would consult their own welfare and find variety in occasionally substituting De Kuyper's Hollands for the more usual form of spirit.

### SUGAR-FREE PALE ALE.

(Harvey and Co, Pellon Brewery, Halifax.)

The above firm are now brewing a very excellent light beer specially adapted to the requirements of persons whose constitution does not allow of their partaking of saccharine beverages. In quality and taste this beer strongly resembles the German lager beers which are imported into this country and find much favour with the gouty and rheumatic. As its title implies, this beer is devoid of free sugar, it has an excellent bitter flavour, is evidently carefully brewed, and is highly to be recommended to the large class of persons who like their glass of beer, but fear to indulge their fancy at the cost of an attack of one of the many gouty manifestations which so frequently originate from the imbibition of saccharine beverages.

